# Synthesis and Characterization of High-Efficiency Halide Perovskite Nanomaterials for Light-Absorbing Applications

**DOI:** 10.1021/acs.iecr.2c00416

**Published:** 2022-04-25

**Authors:** Ahmed Waseem Faridi, Muhammad Imran, Ghulam Hasnain Tariq, Sana Ullah, Syed Farhan Noor, Sabah Ansar, Farooq Sher

**Affiliations:** † Department of Physics, Khwaja Fareed University of Engineering and Information Technology, Rahim Yar Khan 64200, Pakistan; ‡ Department of Mechanical Engineering, Khwaja Fareed University of Engineering and Information Technology, Rahim Yar Khan 64200, Pakistan; § Center of Excellence in Solid State Physics, University of the Punjab, Lahore 54590, Pakistan; ∥ Department of Clinical Laboratory Sciences, College of Applied Medical Sciences, King Saud University, P.O. Box 10219, Riyadh 11433, Saudi Arabia; ⊥ Department of Engineering, School of Science and Technology, Nottingham Trent University, Nottingham NG11 8NS, United Kingdom

## Abstract

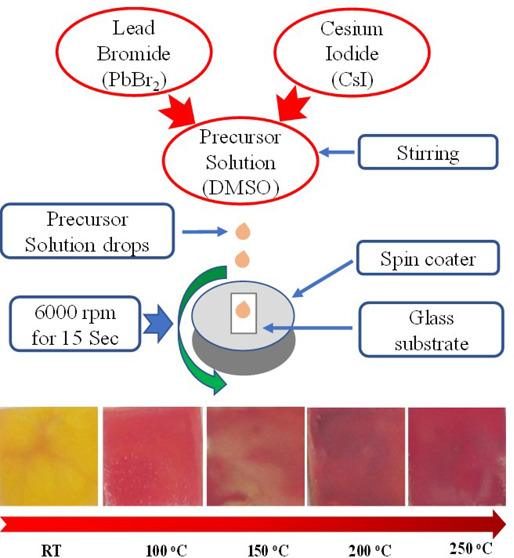

Inorganic perovskite materials are possible candidates for conversion of solar energy to electrical energy due to their high absorption coefficient. Perovskite solar cells (PSCs) introduced a new type of device structure that has attention due to better efficiencies and interest in PSCs that has been increasing in recent years. Halide perovskite materials such as CsPbIBr_2_ show remarkable optical and structural performance with their better physical properties. Perovskite solar cells are a possible candidate to replace conventional silicon solar panels. In the present study, CsPbIBr_2_ perovskite materials’ thin films were prepared for light-absorbing application. Five thin films were deposited on the glass substrates by subsequent spin-coating of CsI and PbBr_2_ solutions, subsequently annealed at different temperature values (as-deposited, 100, 150, 200 and 250 °C) to get CsPbIBr_2_ thin films with a better crystal structure. Structural characterizations were made by using X-ray diffraction. CsPbIBr_2_ thin films were found to be polycrystalline in nature. With increasing annealing temperature, the crystallinity was improved, and the crystalline size was increased. Optical properties were studied by using transmission data, and by increasing annealing temperature, a small variation in optical band gap energy was observed in the range of 1.70–1.83 eV. The conductivity of CsPbIBr_2_ thin films was determined by a hot probe technique and was found to have little fluctuating response toward p-type conductivity, which may be due to intrinsic defects or presence of CsI phase, but a stable intrinsic nature was observed. The obtained physical properties of CsPbIBr_2_ thin films suggest them as a suitable candidate as a light-harvesting layer. These thin films could be an especially good partner with Si or other lower band gap energy materials in tandem solar cells (TSC). CsPbIBr_2_ material will harvest light having energy of ∼1.7 eV or higher, while a lower energy part of the solar spectrum will be absorbed in the partner part of the TSC.

## 1 Introduction

Energy demand has increased due to population and industrial growth, while conventional fossil fuel energy sources are limited and also create environmental problems. Reliable alternate energy sources are required to overcome the issues associated with these fossil fuels. Solar energy has become a promising alternate renewable energy candidate as its source is unlimited and does not produce pollutants damaging to the climate. Solar energy can be captured and utilized for different applications with the help of solar cell devices. Different kinds of solar cells are available including copper zinc tin sulfide (CZTS),^1,2^ cadmium telluride (CdTe),^3,4^ dye-sensitized solar cells (DSSCs),^5,6^ and perovskite solar cells,^7^ among other. Inorganic perovskite solar cells got interested due to their relative better stability efficiency.^8^ The type of material known as “perovskite” is attributed to the structure of calcium titanate (CaTiO_3_) has a molecular composition of type ABX_3_. Perovskite materials have generated a lot of attention due to their cubic layer octahedral perovskite structure and unusual thermal, optical, and electromagnetic characteristics.

Perovskite is a word that refers to any substances that have a crystal structure similar to that of calcium titanate (CaTiO_3_) or comparable to that of calcium titanate (CaTiO_3_). The fundamental formula for the perovskite materials is (ABX_3_), where B would be a cation of a metal and X would be a halide anion (I^–^, Cl^–^, and Br^–^).^9^ Perovskite materials have a long history dating back to 1839, when a German scientist called Gustav Rose found a unique calcium titanate based mineral in the Ural Mountains and termed it “perovskite” after Lev von Perovski, a Russian mineralogist. In 1978, Weber’s group at the University of Stuttgart in Germany created the first inorganic–organic halide perovskite, substituting the cesium cations. In 2009, Miyasaka and his colleagues became the first to employ perovskite-structured materials in solar cells, they utilized CH_3_NH_3_PbI_3_ and CH_3_NH_3_PbBr_3_ as inorganic–organic hybrid halide-based perovskites, to replace the dye component in dye-sensitized solar cells (DSSCs). They achieved relatively low power conversion efficiencies (PCEs) of 3.13 and 3.81%, respectively.

Following this accomplishment, PSC research grew increasingly popular in the field of photovoltaic (PV) research over the next few years. PSC efficiency was eventually increased to 22.1% in early 2016.^10^ PSCs have attracted attention as a promising photovoltaic technology showing high efficiency and low manufacturing cost.^11,12^ However, management for waste with toxic lead (Pb) has not developed yet.^13^ The PSCs efficiency has improved from 14.1 to 25.2% in the last 6 years, making it the third greatest single-junction efficiency ever reported.^6^ In PSCs using CuInS_2_/carbon hole collector and mesoporous TiO_2_ layers as an electron transport layer (ETL), an efficiency of 16.1–16.3% was obtained.^14^ Initially, the perovskite-based “dye” was not a stable “dye”. The perovskite was proven to absorb light better than dye molecules (N719), but the device was damaged after 10 min when oxidation appeared in the liquid electrolyte. A solid-state hole-transport material was added to prevent this degradation, and the system’s efficiency was greatly boosted.^15^

These upgrades incorporated performance improvements from thin-film PVs and DSSCs, as well as other research. In the absence of (TiO_2_) support, solar cells were constructed similarly to thin film PVs, with perovskite acting alone as a light absorber. Planner PSCs were successfully created with a 1.8% efficiency.^16^ Although in another work, researchers retained TiO_2_ the charge blocking layer after changing the conditions of perovskite synthesis and increasing output to 11.4%. Mesoscopic and planar framework solar cells have efficiencies of 20.8 and 21.6%, respectively.^17^ Charge creation (formation of exciton by light absorption), charge transit, recombination, and extraction are all working mechanisms^18^ in these devices. Perovskite materials have a long charge diffusion length, a high absorption coefficient and high charge mobility and low exciton (e^–^–h^+^ pair) binding energy, making perovskite materials an excellent candidate for use as an active layer in solar cells.

Furthermore, the high solubility of the precursor materials and the low cost of solution processing for the synthesis of perovskite materials, the perovskite solar cells are more cost-effective than silicon solar cells. Another preference is the tunable band gap of perovskite materials, which is relatively adjustable (e.g., MAPb_*x*_Sn_1–*x*_Br_3_ single crystals were obtained with a tunable band gap (2.18–1.77 eV).^19^ Also, perovskite materials have a broad absorption coefficient of >10^4^ cm^–1^, comparable to other solar cell thin film materials like (CZTS) and (CdTe). Perovskite materials have a unique benefit in that they have a high tolerance for flaws and the flexibility to change the material’s composition and physical properties suit for their applications.^20,21^ Flexible perovskite solar cells (FPSCs) are the most favorable for the commercialization option of this technology because the FPSCs can be prepared by applying a roll-to-roll printing procedure for mass production.^22,23^ This technology has become promising for next-generation, high-efficiency, low-cost photovoltaics for multijunction tandem cell structures.^24^

Perovskite materials that are known as halide perovskite materials have halogens (i.e., iodide (I^–^), chloride (Cl^–^), or bromide (Br^–^)) as a constituent part of them. CsPbI_3_ and CsPbI_2_Br are two extraordinary semiconductor materials that can be utilized in optoelectronic detectors like usable layers.^25^ There are optimum band gaps and limited exciton binding energies of optoelectronic perovskite materials like CsPbIBr_2_.^26^ Perovskite materials containing halide group elements were identified in the last two decades as among the most potential material in photovoltaic and other optoelectronic devices. This has contributed to significant developments in materials science, but the word “perovskite” has also caused general confusion and misuse. A perovskite that comprises heavier halides, both entirely inorganic and hybrid inorganic–organic halides, became much more interesting (Cl, Br, and I).^27^ Despite the increase in the PCEs of PSCs from 3.8 to 25.5% over 10 years of research, however, the outdoor applications of PSCs are still significantly limited by poor stability PSCs devices^28−30^ and lead (Pb) toxicity.^31^ Perovskite thin films can be deposited by different methods^32^ including ambient spray coating,^33^ chemical vapor deposition,^34^ ambient deposition,^35^ solution processing,^36^ RF magnetron sputtering,^37^ one-step solution deposition,^38^ blade coating,^39,46^ thermal evaporation,^40−42^ dip coating,^43^ chemical bath deposition,^44^ and electrodeposition.^45^

To the best of our knowledge, detailed studies are not available on the improvement of physical properties of CsPbIBr_2_ thin films by annealing also determination of conductivity type is not available. In the present study, inorganic halide perovskite thin films were prepared by one-step solution deposition technique using a spin-coating method. The deposited thin films were annealed at different temperatures to optimize their structure because the structure itself will determine all the other physical properties related to their applications. Furthermore, these are characterized to achieve optimum physical properties for applications as the light-absorbing layer in solar cells. The obtained physical properties of CsPbIBr_2_ thin films suggest them a suitable candidate as a light-harvesting layer for future solar cell applications.

## 2 Experimental Section

### 2.1 Materials

Cesium iodide (CsI) and lead bromide (PbBr_2_) as primary precursors, as well as dimethyl sulfoxide (DMSO), isopropanol (IPA), acetone, and distilled water as solvent and cleaning solutions, were used to synthesize CsPbIBr_2_ thin films. All chemicals were purchased from Sigma Company. Microscopic glass slides were used as substrates.

### 2.2 Substrate Cleaning

Substrate cleaning is an important part of an experiment before thin film deposition. A well-cleaned contaminations free substrate is necessary not only for good adhesion but also for contaminations’ free deposited thin films. The glass substrates were cleaned with a detergent for 5 min, and after this substrate is washed with distilled water. Next, the glass substrate was put in IPA and acetone for 5–10 min, respectively, and then dried at 60 °C. All cleaned substrates were kept in IPA before using for deposition.

### 2.3 Deposition of CsPbIBr_2_ Thin Films

Traditional one-step processing via a spin-coating method^38^ was adopted for CsPbIBr_2_ thin films on cleaned glass substrates. Precursor solution was prepared by taking CsI (600 mg), PbBr_2_ (800 mg), and 2 mL of DMSO solvent. First, PbBr_2_ was dissolved in DMSO (2 mL) until PbBr_2_ was completely dissolved in the solvent, then this solution was placed on a hot plate and stirred at 70 °C for 30 min to prepare a uniform solution. Then, 600 mg of CsI was added to prepare the solution of CsPbIBr_2_, and stirring was continued at 70 °C for 30 min. When the precursor solution was obtained, the CsPbIBr_2_ thin film was deposited on a clean glass substrate by placing the substrate on a spin-coater and setting the program at 6000 rpm for 15 s. Then the prepared solution of CsPbIBr_2_ was placed on the glass substrate by dropper, and the spin-coater was run at 6000 rpm for 15 s.

### 2.4 Annealing of CsPbIBr_2_ Thin Films

**Figure 1:**
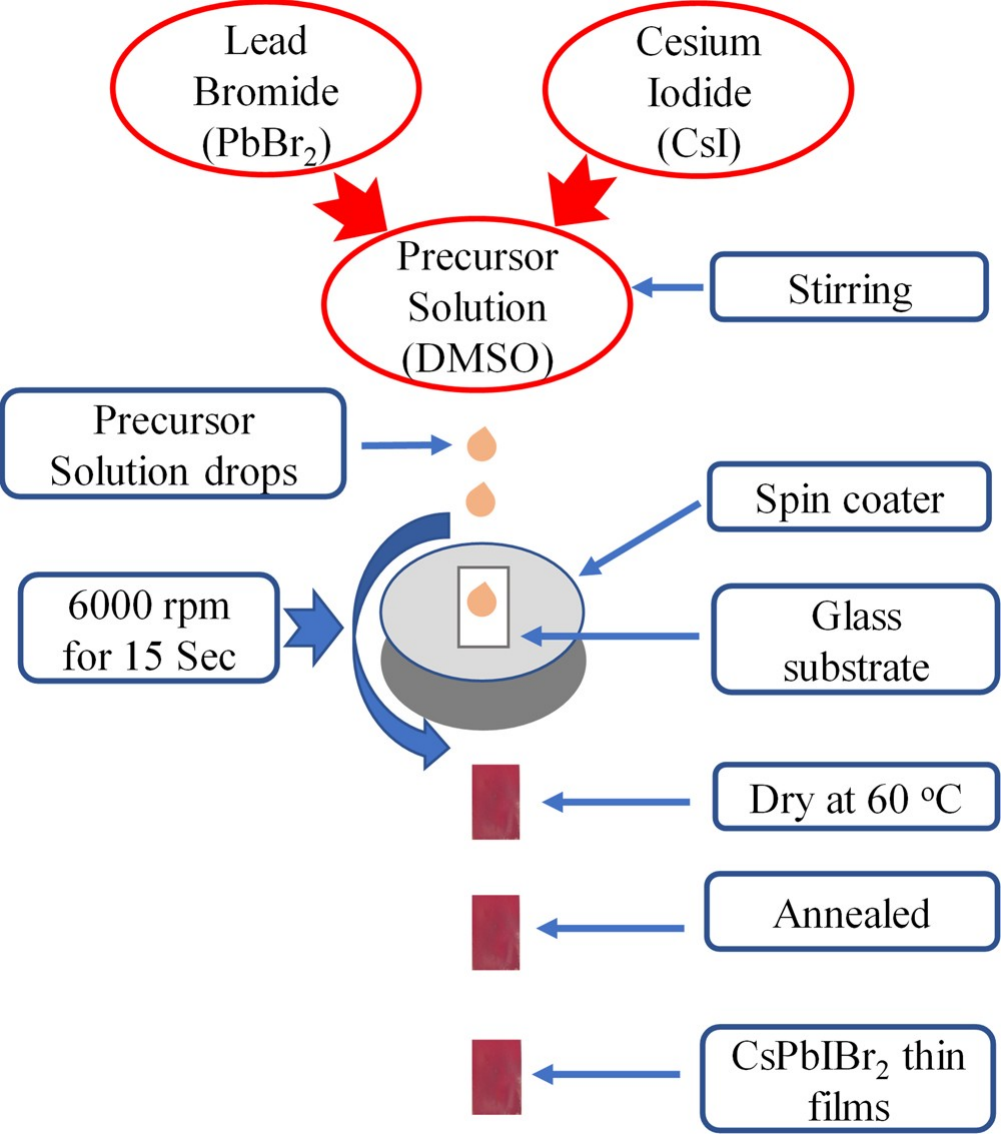
Experimental work flowchart for the preparation of precursor solution and deposition of CsPbIBr_2_ thin films.

**Figure 2:**
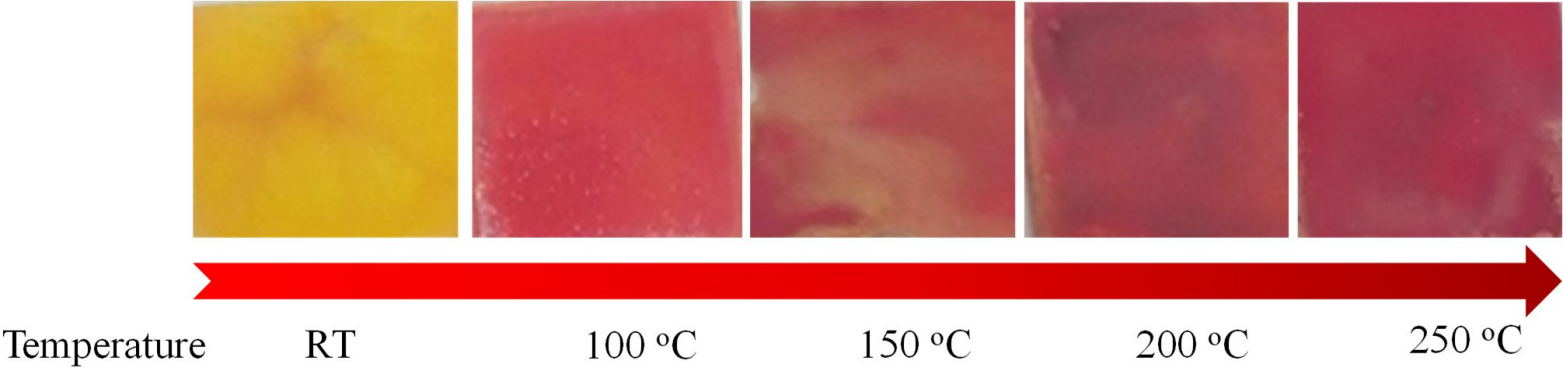
CsPbIBr_2_ thin films prepared at different annealing temperatures.

The deposited thin films were allowed to dry for 5 min at 60 °C in the oven. Furthermore, these deposited thin films were annealed at different temperatures (100, 150, 200, and 250 °C) to improve the structure of CsPbIBr_2_ thin films, one sample was left as deposited (i.e., at room temperature (RT)). Finally, these annealed CsPbIBr_2_ thin films were kept in an airtight box for characterization. The experimental procedure is shown in Figure 1, and pictures of the prepared CsIPbBr_2_ thin films annealed at different annealed temperatures are shown in Figure 2.

### 2.5 Characterization

The thickness of prepared CsPbIBr_2_ thin films was estimated with the help of the gravimetric weight different method^44,45^ by adopting the following steps: (1) measured the mass (*m*_1_) blank substrate i.e., before deposition, (2) measured the mass (*m*_2_) after deposition of the CsPbIBr_2_ thin film on the substrate, and (3) calculated the mass difference (*m* = *m*_2_ – *m*_1_), which denotes the mass of deposited thin film on the substrate. Then the estimated thickness of thin films was calculated according to the equation *t* = *m*/(ρ × *A*),^45^ where *m* is the mass of thin film deposited on the substrate, *A* is the area of the deposited thin film in cm^2^, ρ is the average density of the CsPbIBr_2_ = 4.44 g/cm^3^, and *t* is the thin film thickness. The calculated thickness was about 450 nm of all deposited thin films.

## 3 Results and Discussion

Characterizations to find the structural parameters (i.e., crystalline nature, crystallite size, and microstrains) of CsPbIBr_2_ thin films were done by using X-ray diffraction (XRD) with Cu Kα X-ray radiations (λ = 1.5406 Å) in the diffraction angle (2θ) scan range 20–60°. Optical properties (i.e., absorption coefficient and band gap energy) were calculated from transmission data acquired by UV–vis spectroscopy in the wavelength range of 250–1000 nm. The conductivity type of prepared CsPbIBr_2_ thin films was identified by applying a hot-probe (two-probe) technique. These characterizations were made within 3 weeks after the preparation of CsPbIBr_2_ thin films.

### 3.1 Structural Analysis

**Figure 3:**
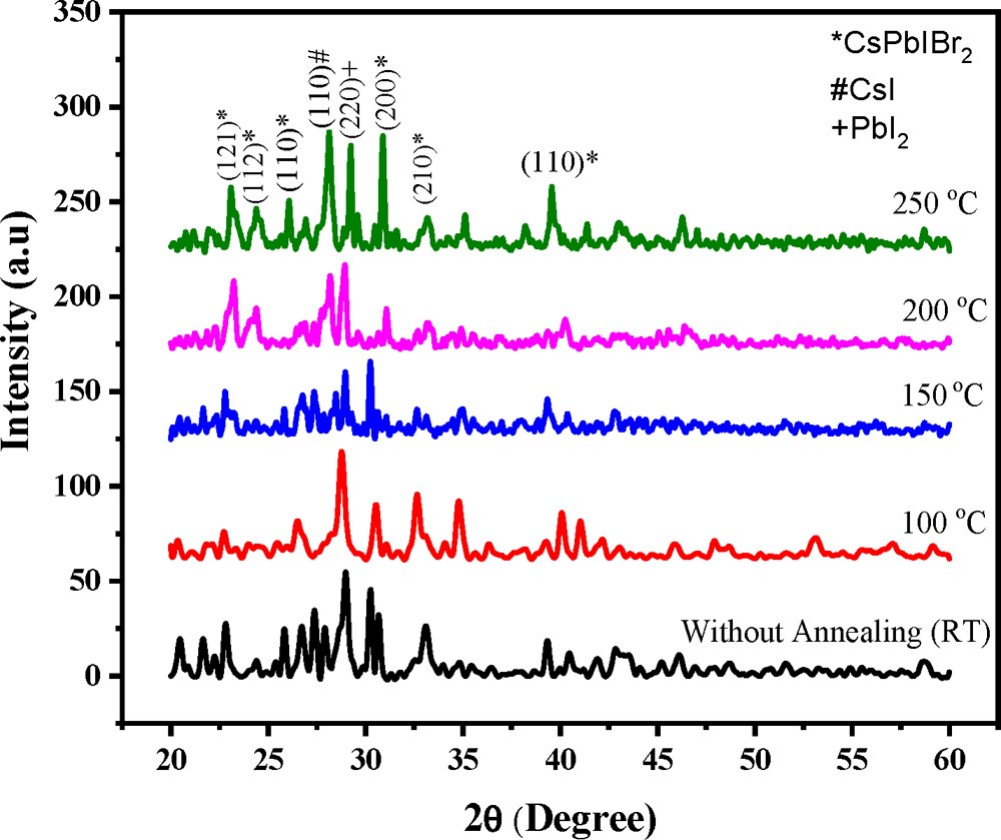
XRD spectra of CsPbIBr_2_ thin films prepared at different annealing temperatures.

**Figure 4:**
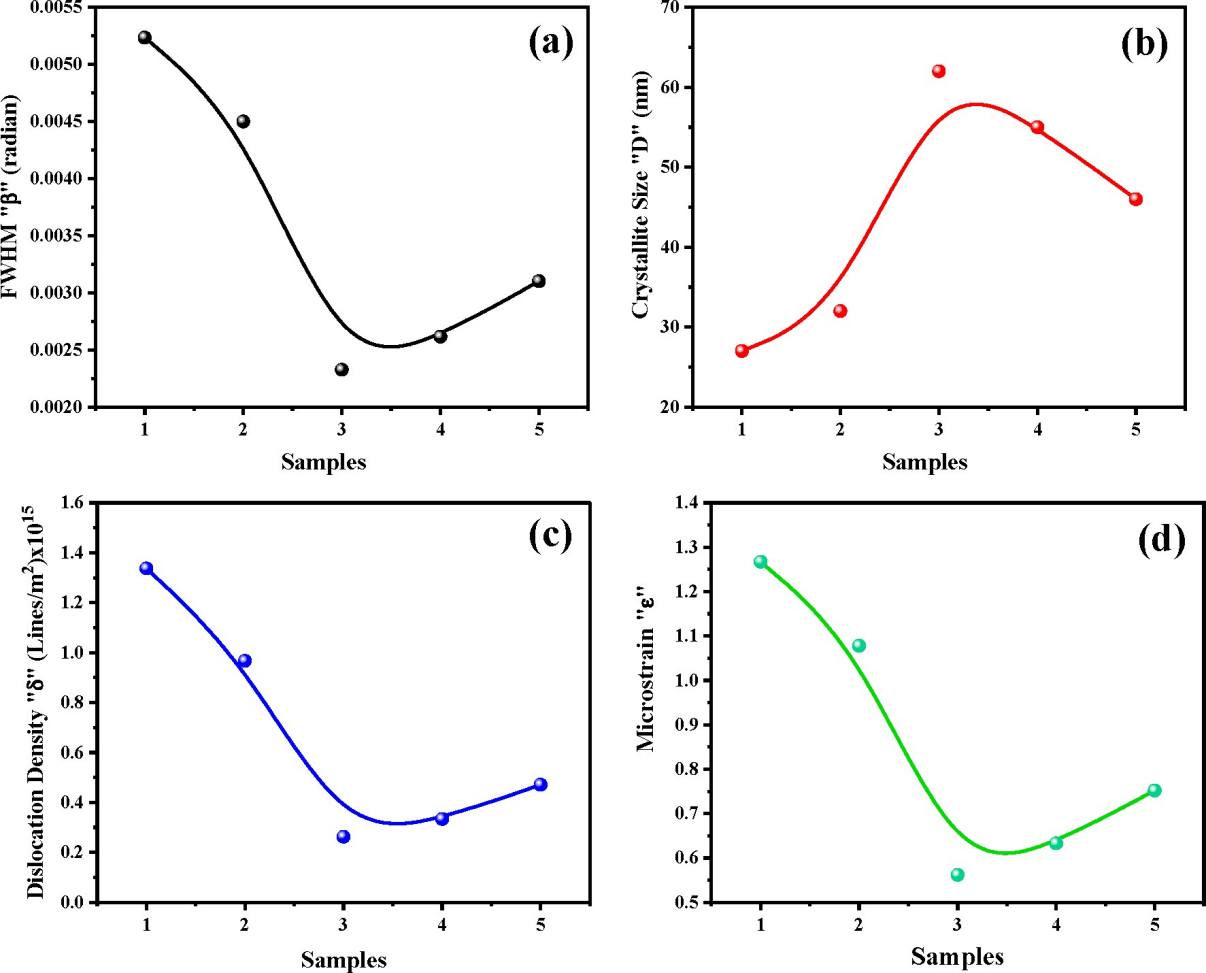
Dependence on structural parameters (a) β, (b) *D*, (c) δ, and (d) ε of CsPbIBr_2_ thin films prepared at different annealing temperatures.

**Figure 5:**
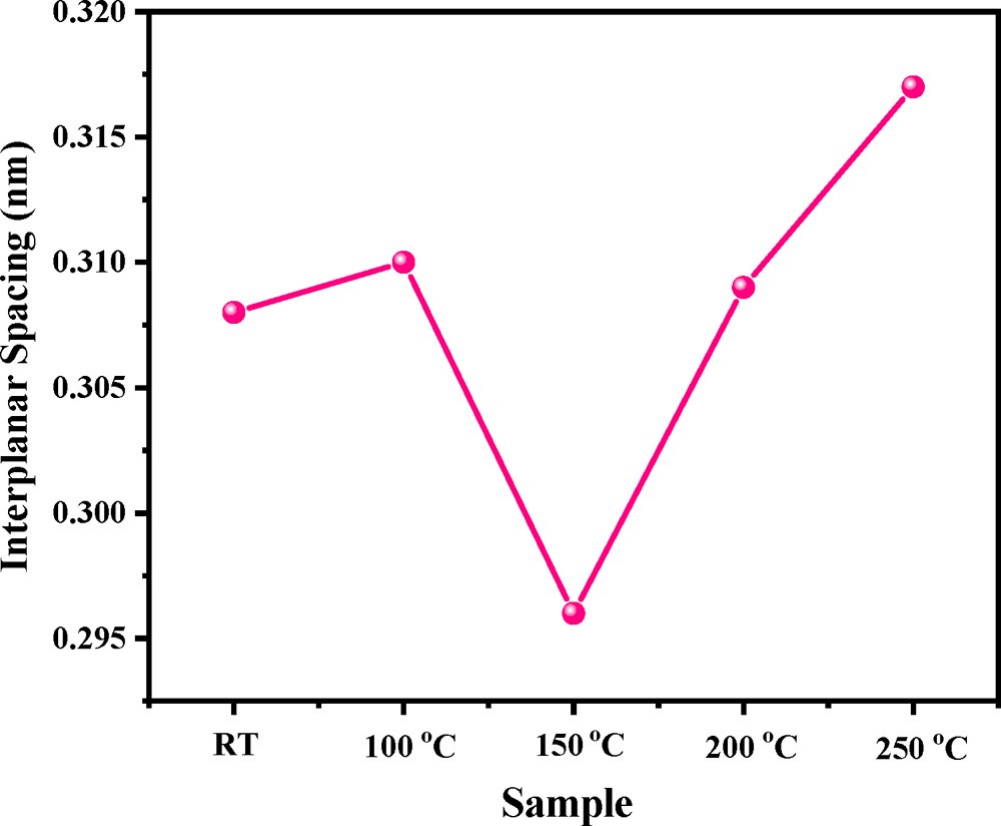
Dependence of interplanar spacing of CsPbIBr_2_ thin films prepared at different annealing temperatures.

XRD is a frequently used technique for structural characterization and to identify the crystalline nature of prepared samples. XRD spectra obtained for all samples are shown in Figure 3. These spectra showed that the CsPbIBr_2_ thin films deposited on glass substrates are polycrystalline in nature. The diffraction peaks of CsPbIBr_2_ thin films are observed at the diffraction angles (2θ) 23.10, 24.36, 26.10, 30.91, 33.14, and 39.50° belonging to crystallographic planes’ orientations (121), (112), (110), (200), (210), and (110), respectively; while peaks at 28.16 and 29.17° could be assigned to planes (110) CsI, and (220) PbI_2_, respectively.^29,30^ Furthermore, these XRD spectra revealed that by increasing the annealing temperature the crystallinity of CsPbIBr_2_ thin film is improved. From the spectra data, crystallite size *D* (nm) was calculated by using a well-known Scherrer formula^47^ as given in eq 1.
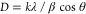
1where *D*, λ, θ, β, and *k* are the crystalline size, wavelength, angle of diffraction, full width half-maximum (fwhm), and Scherrer constant (*k* = 0.9), respectively. Microstrain was calculated by using the following eq 2.^48^ Dislocation density σ (lines per meter square) was calculated by using eq 3.^48^
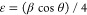
2
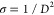
3where *D* is the crystallite size. The obtained crystalline size, microstrain, dislocation density, and *d*-spacing are presented in Table 1. Initially, the crystalline size of the first sample is about 27.35 nm which is for the sample left as prepared and without annealing. After that crystalline size is increased with an increase in the annealing temperature, but at 200 °C annealing temperature, the crystalline size was decreased by increasing the annealing temperature which might be due to recrystallization. The variation of crystallite at 200 and 250 °C may be connected to the growth of CsPbIBr_2_ at different orientations. However, microstrain and dislocation density were also found to be annealing-temperature-dependent; the obtained values are presented in Table 1 and shown in Figure 4, which predicted that with the growth of crystallite size both microstrain and dislocation density were smaller Interplanar spacing (*d*) was found by using the well-known Bragg’s law, and obtained values are given in Table 1. The trend depending on annealing temperature is shown in Figure 5. The interplanar spacing is initially decreased, but on higher annealing temperature, it is increased which showed the structural dependence of thin films on the annealing temperature. This variation in interplanar spacing value is due to crystallite size, as when crystallite size is larger the interplanar spacing is smaller which may be due to stronger interatomic bonding.^49,50^

**Table 1:** XRD Structural Parameters of CsPbIBr_2_ Thin Films Annealed at Different Temperatures

sample	fwhm (2θ) radian	crystallite size (nm)	dislocation density (δ) × 10^15^ (linens/m^2^)	microstrains (ε) × 10^3^	interplanar spacing (*d*) (nm)
CsPbIBr_2_-1	0.005234	27	1.337	1.267	0.308
CsPbIBr_2_-2	0.004500	32	0.967	1.078	0.310
CsPbIBr_2_-3	0.002326	62	0.263	0.562	0.296
CsPbIBr_2_-4	0.002616	55	0.334	0.633	0.309
CsPbIBr_2_-5	0.003101	46	0.471	0.752	0.317

### 3.2 Optical Properties

**Figure 6:**
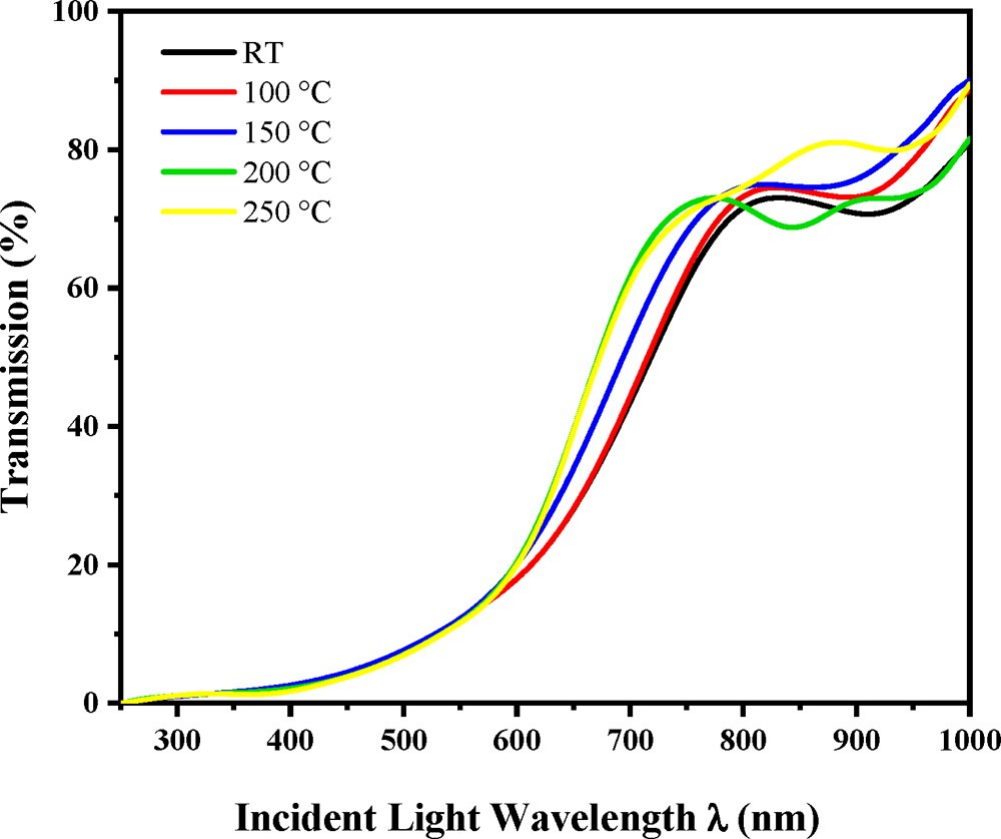
Transmission spectra of CsPbIBr_2_ thin films annealed at different temperatures.

**Figure 7:**
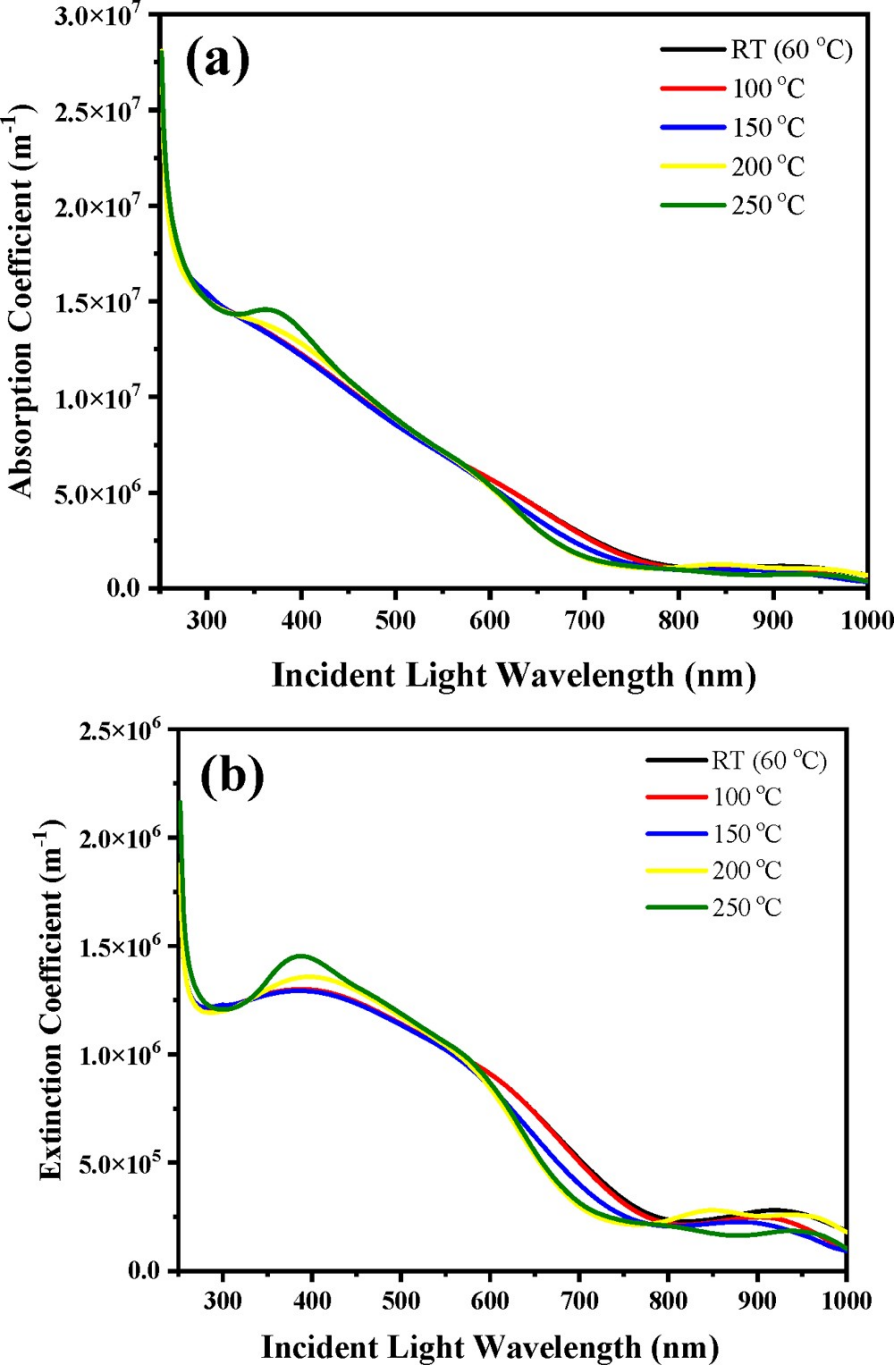
Dependence effect. (a) Absorption coefficient. (b) Extinction coefficient of CsPbIBr_2_ thin films prepared at different annealing temperatures.

Optical properties were studied by obtaining the transmission spectra between 250 and 1000 nm by using UV–vis double beam spectrophotometer. The obtained transmission spectra for all CsPbIBr_2_ prepared thin films are shown in Figure 6, all samples showed a transmission of lower than 40% in the visible region. The absorption coefficient of prepared CsPbIBr_2_ thin films was found from obtained transmission data by using the relation,^51^ given in eq 4. The extinction coefficient *k* of CsPbIBr_2_ thin films was calculated from eq 5.^52^
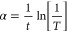
4
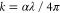
5where *t* is thickness of thin films and *T* is percent transmission. Obtained values of absorption coefficient were used to find the band gap energy. The study revealed that the absorption edge is shifted at higher values of incident light energies (i.e., a blueshift is observed). Generally, the blueshift in thin film materials may be associated with the Burstein–Moss effect.^53^ The dependence of absorption coefficient and extinction coefficient on annealing temperature is plotted in Figures 7a,b, respectively, which show the dependence of absorption and extinction coefficients on wavelength λ of incident light. The observed variations in the extinction coefficient for the higher energies may be due to the band-to-band transition of charge carriers.^54^ However, for lower energies of the incident light, *k* values are observed comparatively smaller that allowing the incident light to pass through the thin films several times.

**Figure 8:**
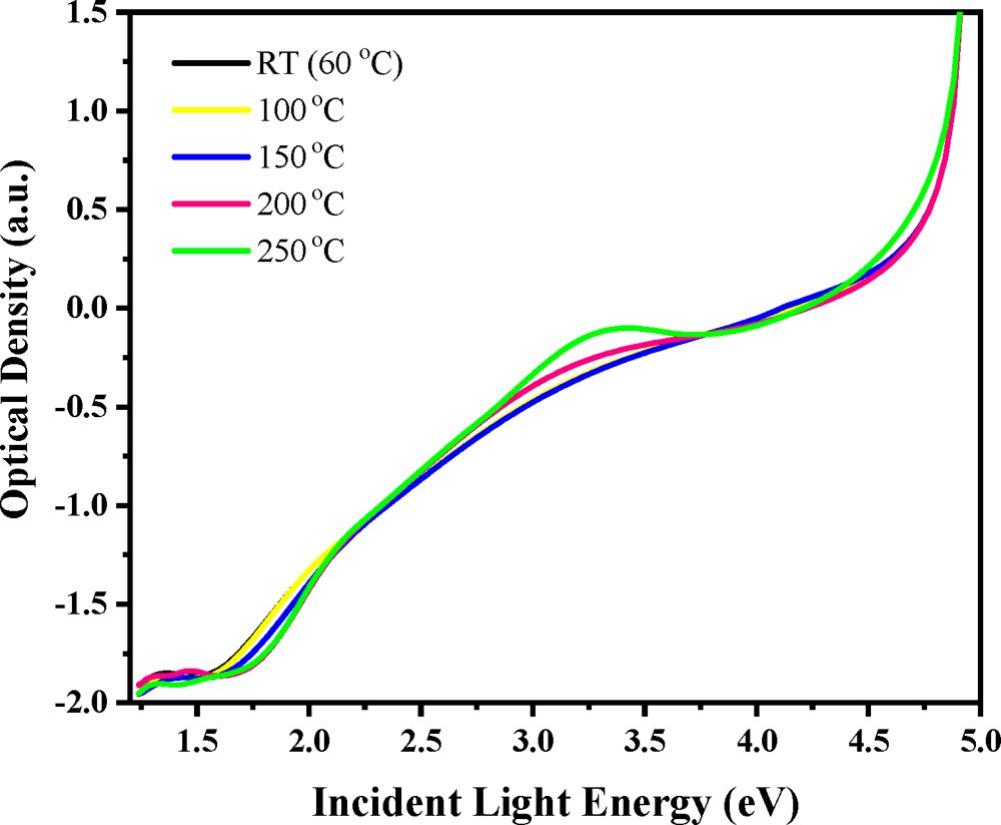
Annealing temperature dependence of optical density for CsPbIBr_2_ thin films.

Therefore, in the transmission region, interference fringes are produced, as shown in Figure 6. Moreover, Figure 7a shows that all prepared samples are capable of efficiently absorbing the incident light in UV–visible region which is well in agreement with findings in literature for CsPbIBr_2_ thin films,^55^ and Figure 7a also shows that the samples annealed at higher annealing temperatures have higher absorption. Figure 7b demonstrates the variation in extinction coefficient *k* due to the postdeposition annealing temperatures of CsPbIBr_2_ thin films and depicted that the value of extinction coefficient *k* for these thin films became higher with increasing the annealing temperature. For an incident light wavelength of λ = 350 nm, the absorption and extinction coefficients are higher, while these coefficients decrease as λ increases and gradually diminishes. The optical density (OD) of CsPbIBr_2_ thin films was also calculated, which is the measure of transmittance of an optical medium at a specific wavelength that was calculated from the obtained transmission data with the help of relation given in eq 6.^56^
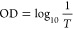
6where *T* is the transmission. The dependence of OD on energy of incident light is shown in Figure 8, which illustrates the variation in optical density with wavelength λ of incident light. The OD shows the same dependence as that of the absorption coefficient, which indicates the increase in absorption ability of CsPbIBr_2_ thin films for incident light.

### 3.3 Determination of Band Gap Energy (*E*_g_)

**Figure 9:**
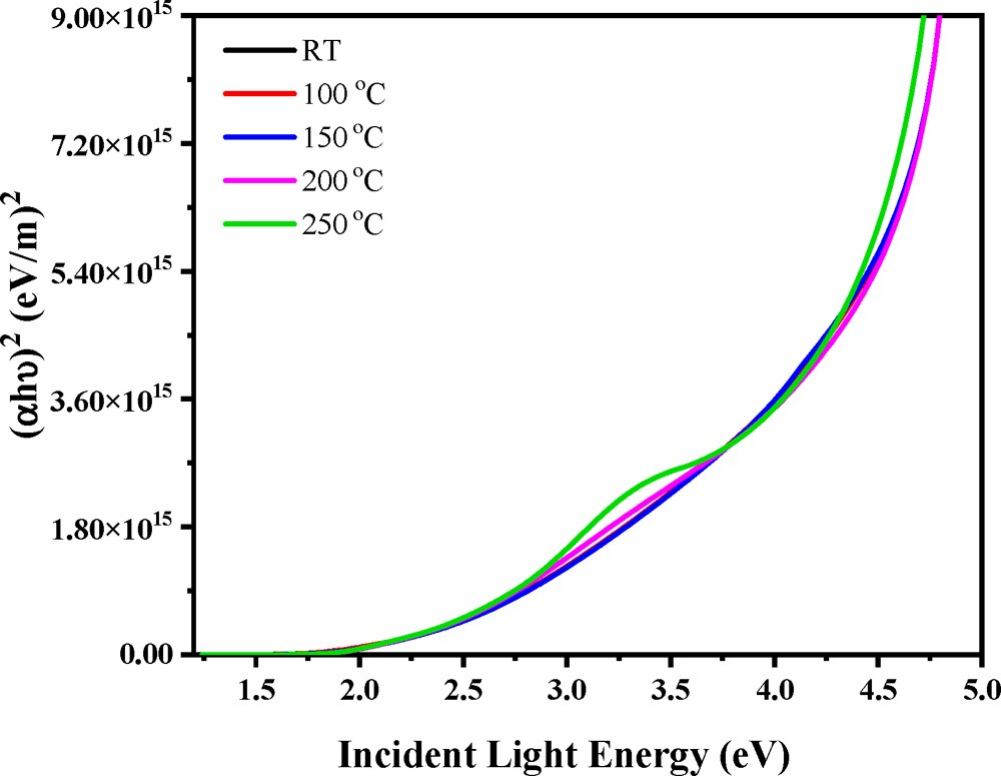
Plots of (α*h*ν)^2^ against *h*ν of CsPbIBr_2_ thin films annealed at different temperatures.

**Figure 10:**
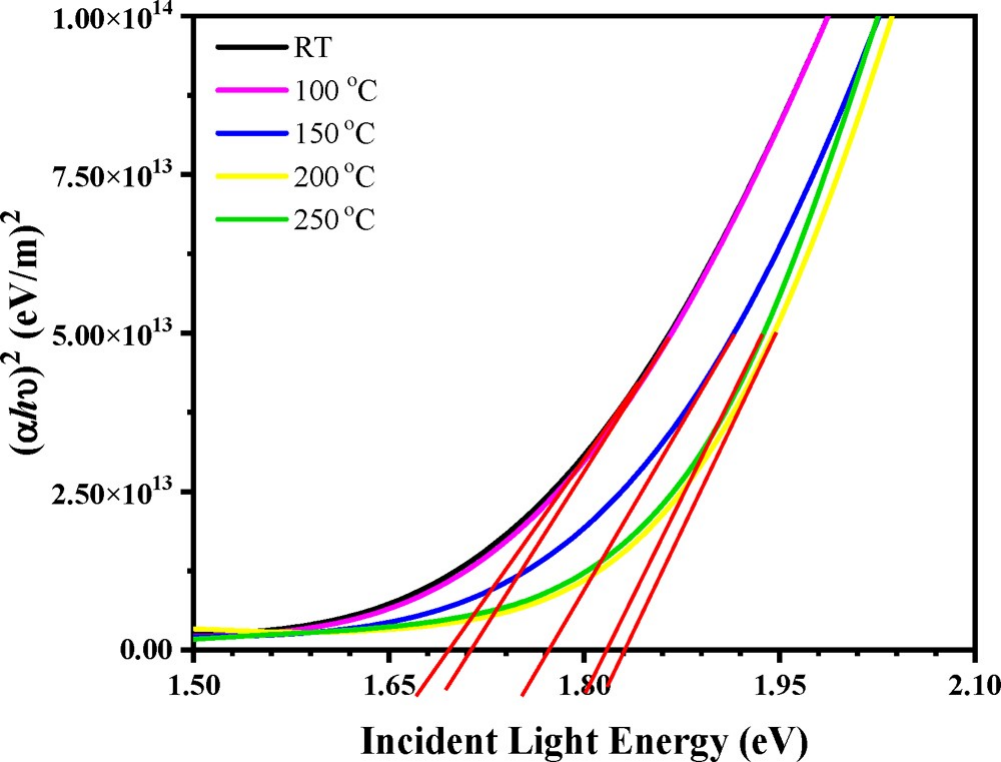
Linear part of plots near absorption edge extrapolated to energy axis for CsPbIBr_2_ thin films.

Halide perovskite semiconductors have an optimum band gap to harvest the maximum part of the incident solar energy spectrum. Mixing/interchanging B cations (Sn, Pb, and Ge) or halogens (X = Br, I, and Cl) may adjust the band gap of halide perovskite from 1.1 to 3.7 eV.^57^ Halide perovskites are semiconductors with a direct band gap and strong absorption edges. Figure 7a reveals that by changing the annealing temperature, there is a slight change in the absorption coefficient. It measures how much light is absorbed by the material at a specific wavelength of the incident light, which depends on the energy gap present between the valence and conduction bands of CsPbIBr_2_. The band gap energy (*E*_g_) of CsPbIBr_2_ thin films was calculated with the help of Tauc relation^58^ (eq 7).
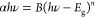
7where α, *h*, ν, and *n* are the absorption coefficient, Plank’s constant, the frequency of the incident light, and a constant number, respectively. For a direct transition of electron, (*n* = 1/2) or (α*h*ν)^2^ = *B*(*h*ν – *E*_g_), which results in a linear relation between (α*h*ν)^2^ and light energy (*h*ν). In the present work, plots of (α*h*ν)^2^ versus the incident light energy (*h*ν) were plotted as seen in Figure 9. All these plots have a linear behavior for high energy incident light. This indicated that the Tauc relation holds good for CsPbIBr_2_ thin films if *n* = 1/2. This observation confirmed the direct transition of electrons for these CsPbIBr_2_ thin films prepared at different annealing temperatures. The value of the band gap energy (*E*_g_) was found by extrapolating the linear part of the absorption plot (*αhν*)^2^ and its intercept to the *x*-axis (i.e., *h*ν-axis), where the absorption coefficient (α = 0) is shown in Figure 10. A blueshift is observed in the absorption edge that is due to an increase in band gap energy with increasing annealing temperature.

**Figure 11:**
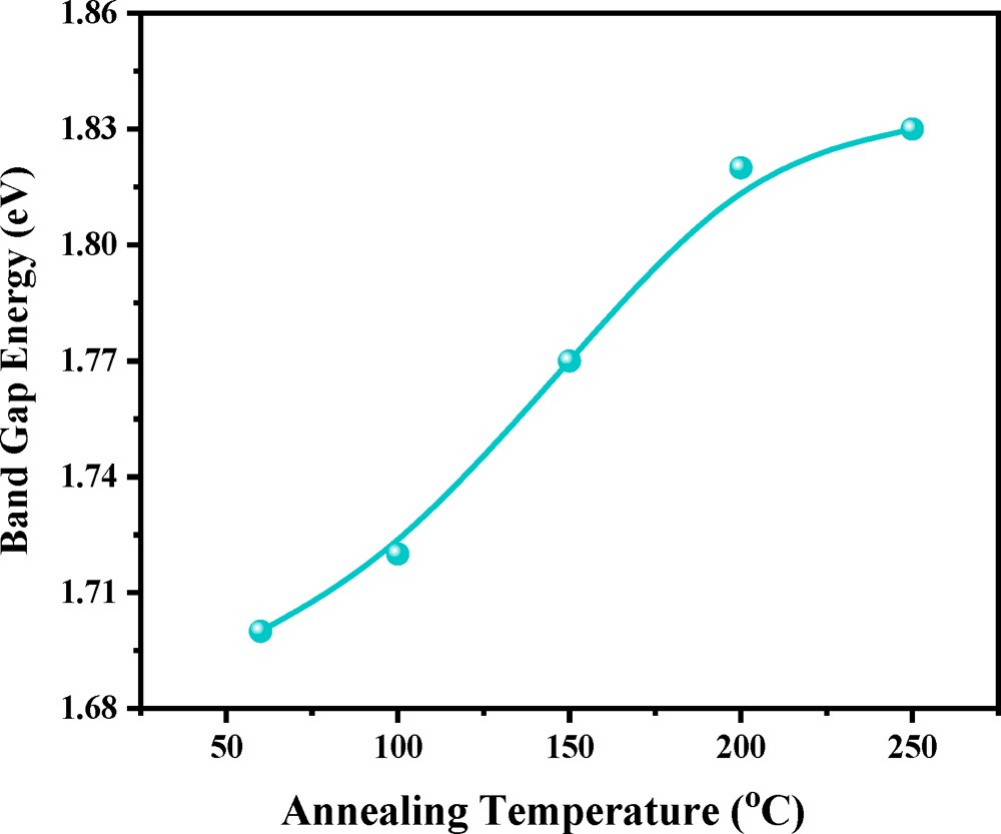
Variation in band gap energy values of CsPbIBr_2_ thin films with annealing temperature.

The obtained values of the band gap range from 1.70 to 1.83 eV for CsPbIBr_2_ thin films annealed at (as-deposited, 100, 150, 200 and 250 °C) respectively that could be ascribed annealing induced structural transformations in the deposited CsPbIBr_2_ thin films. These obtained values of band gap energy are given in Table 2. The variation in band gap energy values for different annealing temperatures is plotted in Figure 11; it shows that with increasing annealing temperature the band gap energy is increased, which could be attributed to the variation in structural properties of prepared thin films. The obtained values of band gap energy are comparable with the literature.^59^ These findings are also inconsistent with the previously reported value (2.05 eV) for CsPbIBr_2_ thin films.^60^

**Table 2:** Band Gap Energy of CsPbIBr_2_ Thin Films Prepared at Different Annealing Temperatures

sample	annealing temperature (°C)	band gap energy *E*_g_ (eV)
1	RT	1.70
2	100	1.72
3	150	1.77
4	200	1.82
5	250	1.83

### 3.4 Conductivity Type Determination

The conductivity type of semiconductor can be checked with different methods such as rectification, Hall effect, and thermal electromotive force (emf). The conductivity type of CsPbIBr_2_ thin films we checked by using a thermal emf method (i.e., the hot-probe method).^61,62^ Two probes were placed on the surface of the prepared CsPbIBr_2_ thin films. One probe was heated, while the second probe was kept cold. Before these hot-probe measurements were calibrated with n-type standard Si wafer, the CsPbIBr_2_ thin films did not show any prominent response during applying heat to the probe assigned for heating. There was a little fluctuation opposite to the reference n-type wafer but not a stable reading that we could note and assign the p-type conductivity. This poor fluctuating response toward p-type nature may be due to intrinsic defects or presence of CsI phase. Overall, it was confirmed that the intrinsic nature of prepared samples is inconsistent with the basic nature of perovskite materials.

## 4 Conclusion

The present study is focused on the preparation of CsPbIBr_2_ thin films for light-absorbing layer applications in perovskite solar cells. Precursor solutions of CsI and PbBr_2_ were used, and CsPbIBr_2_ thin films were successfully deposited on glass substrates by one-step spin coating method. We carried out the studies of annealing effects on physical properties and the prepared CsPbIBr_2_ thin films were annealed at different temperatures 100, 150, 200, and 250 °C keeping one sample unannealed for reference. Obtained XRD peaks revealed that the prepared thin films composed of CsPbIBr_2_ perovskite material accompanying some peaks related to CsI and PbI_2_ phases. The Scherrer formula determines the crystalline size (*D*), which showed an increase (27 and 62 nm) with annealing temperatures of 100 and 150 °C, but then some decrease of 55 and 46 nm, respectively, is observed on further increase in annealing temperature up to 200 and 250 °C, respectively. Overall, crystallinity is improved. The optical transmission graphs showed that most of the light absorption in CsPbIBr_2_ thin films occurs in the visible region of the solar spectrum, which is optimum for a solar cell to absorb the most intense part of the solar spectrum. On increasing the annealing temperature, a small blueshift is observed in the absorption edge, and the band gap energy *E*_g_ of CsPbIBr_2_ thin films is shifted from 1.70 to 1.83 eV, which enables them to absorb more parts of the solar spectrum. The conductivity type of these thin films was determined by using a hot-probe method, which showed a weaker fluctuating response toward a p-type nature which may be due to intrinsic defects or the presence of CsI phase; overall, the intrinsic nature of the prepared samples was determined. The obtained physical properties of CsPbIBr_2_ thin films are favorable for their application as light-absorbing layers in photovoltaic applications. However, some more advanced studies are required such as stability of CsPbIBr_2_ thin films in an open environment and long exposure to sunlight. In the future, preparation parameters will be modified for phase stability of CsPbIBr_2_ thin films, and efforts should be focused to improve thin film’s quality to avoid the growth of any additional phase and energy loss of incident light.
